# Tuberculous meningitis in Denmark: a review of 50 cases

**DOI:** 10.1186/1471-2334-11-47

**Published:** 2011-02-22

**Authors:** Anne-Sophie H Christensen, Åse B Andersen, Vibeke Ø Thomsen, Peter H Andersen, Isik S Johansen

**Affiliations:** 1 Department of Infectious Diseases, Rigshospitalet, University of Copenhagen, Copenhagen, Denmark; 2 The International Reference Laboratory of Mycobacteriology, Statens Serum Institut, Copenhagen, Denmark; 3 Department of Epidemiology, Statens Serum Institut, Copenhagen, Denmark; 4 Department of Infectious Diseases, Hvidovre University Hospital, Copenhagen, Denmark

## Abstract

**Background:**

Tuberculous meningitis is the most severe manifestation of extrapulmonary tuberculosis with a high mortality rate and a high rate of sequelae among survivors. The aim of this study is to assess the current epidemiology, clinical features, diagnostic procedures, treatment and outcome in patients with tuberculous meningitis in Denmark, a country with a low tuberculosis incidence.

**Methods:**

A nationwide retrospective study was conducted, comprising all patients notified with tuberculous meningitis (TBM) in Denmark from 2000-2008. Medical records were reviewed using a standardised protocol.

**Results:**

Fifty patients, including 12 paediatric patients, were identified. 78% of the patients were immigrants from countries of high tuberculosis endemicity. 64% of all patients had a pre-existing immunosuppressive condition; 10% were HIV positive, 48% were HIV seronegative and 42% had an unknown HIV status. Median symptom duration before admission was 14 days in the Danish patient population and 20 days in the immigrant group. Biochemical analysis of cerebrospinal fluid (CSF) samples revealed pleocytosis in 90% with lymphocyte predominance in 66%. Protein levels were elevated in 86%. The most common findings on neuro-radiological imaging were basal meningeal enhancement, tuberculomas and hydrocephalus. Lumbar puncture was performed on 42 patients; 31 of these specimens (74%) had a positive CSF culture for mycobacteria and 9.5% were smear positive for acid-fast bacilli. The overall mortality rate was 19% and 48% of the remaining patients had neurological sequelae of varying degree.

**Conclusion:**

TBM is a rare but severe manifestation of extrapulmonary TB in Denmark. The clinician must be prepared to treat empirically if the suspicion of TBM has arisen to improve treatment outcome.

## Background

Tuberculosis (TB) of the central nervous system is the most severe manifestation of extrapulmonary TB and constitutes approximately 1% of all new cases annually [1]. Although the incidence of tuberculous meningitis (TBM) is low in high-income countries, it remains one of the most severe and eventually fatal infectious conditions - especially in times of increasing use of immunosuppressive drugs, increased access to transplantation (also for patients from TB endemic countries), changing HIV patterns and increasing prevalence of type II diabetes.

TBM is characterised by a slowly progressing granulomatous inflammation of the basal meninges. This inflammatory reaction can lead to a number of complications, such as hydrocephalus, cerebral vascular infarction, cranial nerve palsy and, if left untreated, death. Rapid diagnosis and initiation of treatment is therefore necessary to reduce the high mortality and severe sequelae associated with the disease. Diagnosing TBM can be difficult as the symptoms are unspecific and mimic those of meningitis caused by other microbiological agents or other cerebrovascular events.

Denmark has a low TB incidence of 6.7/100.000 with 367 notified cases in 2008 [2]; yet, the country has had fluctuations in the annually reported number of TB cases throughout the last 20 years. In 1986 there was an all-time low of 299 new cases [3] and in 2000 there was a peak of 548 new cases [4], equalling an increase of 83% in 14 years. This increase was primarily due to an increase in immigration from high-incidence countries [3,5].

## Methods

To initiate this retrospective study we obtained data on notified TBM cases from the National TB Notification Register between January 2000 and December 2008. The medical records of all patients, including children, with TBM were retrieved from Paediatric Departments and Departments of Infectious Diseases at five major University Hospitals in Denmark. Of note, in Denmark all cases of tuberculous meningitis are treated at university hospitals as required by national guidelines. Permission was granted from the Danish Data Protection Agency.

Demographic data, medical history, clinical presentation at admission, radiological and microbiological data was reviewed along with the clinical course, treatment and outcome. All microbiological data was retrieved from The International Reference Laboratory of Mycobacteriology at Statens Serum Institut.

## Results

### Demography

50 cases of cerebral TB were notified throughout the period; out of these 15 (30%) had tuberculomas. A total of 12 paediatric patients (aged 1-15 years) were found. All results have been divided into two sub-groups - one consisting of native Danish patients, the other of immigrants. The immigrant group of patients in this study includes patients born abroad and their children up to 25 years of age, as well as people from Greenland living in Denmark.

A total of 11 patients (22%) were native Danes, the remaining 39 (78%) were immigrants from varying countries; three from Greenland, three from Ex-Yugoslavia, four from Sub-Saharan African countries with high HIV prevalence (Ethiopia, Uganda, South Africa), 22 from the Eastern Mediterranean region as defined by the World Health Organisation, WHO, (primarily Morocco, Somalia, Pakistan), and seven from Asia (China, India, Indonesia, Vietnam, Cambodia). 13 out of the 15 (87%) tuberculoma patients were immigrants.

There was an equal sex distribution in the two groups. Median age for the Danish patients was 17 years (range 1-52) and for the immigrant group 34 years (range 1-71).

Further demographic data are specified in Table 1.

**Table 1:** Demographic data, clinical presentation and outcome

	Danes (n = 11)	Immigrants (n = 39)
Male sex (%)	6 (54)	18 (46)
Age, median (range), years	17 (1-52)	34 (1-71)
Pre-existing diseases or conditions (%)	6 (54)	26 (67)
HIV infected patients (%)	1 (9)	4 (10)
Symptom duration, median (range), days	14 (3-180)	20 (5-180)
		
General symptoms & signs		
Fever (%)	11 (100)	31 (79)
Headache (%)	5 (45)	31 (79)
Meningeal stiffness (%)	2 (18)	19 (49)
Weightloss (%)	4 (36)	14 (36)
		
Neurological symptoms		
Confusion (%)	6 (54)	20 (51)
Cranial nerve paralysis (%)	4 (36)	14 (36)
Convulsions (%)	1 (9)	7 (18)
		
Extra-cerebral TB manifestations		
Pulmonary TB (%)	6 (54)	17 (44)
Other localizations (%)	1 (9)	9 (23)
		
Delay in treatment, median (range), days	7 (0-30)	3-4 (0-52)
		
Outcome*		
Full recovery (%)	2 (18)	14 (38)
Sequelae (%)	6 (54)	17 (46)
Death (%)	3 (27)	6 (16)

*n = 37 in the immigrant group as two patients were lost for follow-up

Six Danish patients (55%) had pre-existing immunosuppressive diseases or conditions like malignancy or diabetes. Three patients were taking immunosuppressive medication due to organ transplant. Some were recorded as having a drug and/or alcohol abuse, and one patient was known HIV positive at the time of TBM diagnosis.

Similar results were found in the immigrant group with 26 out of 39 patients (67%) having one or more dispositions. Among these patients, four were HIV positive, two had kidney transplants, six had diabetes and four were recorded as having an alcohol abuse. Two patients had a family member who had recently been treated for TB.

Within the two patient populations, a total of 24 patients (48%) were HIV seronegative and 21 had an unknown HIV status (42%).

### Clinical presentation

Over half the patients presented with fever and headache. The classic sign of meningeal stiffness was found in less than half the patients. Neurological signs upon admission was affected in the majority of cases: 52% of the patients were described with a general altered mental state (i.e. confusion), 36% had cranial nerve paralysis (predominantly facial nerve or abducens nerve affection) and 16% presented with generalised convulsions upon hospitalization (Table 1).

Five of the immigrant patients were initially admitted at neurology departments with headaches, convulsions and altered cerebral conditions. Due to symptoms of increased intra-cranial pressure, these patients underwent CT scanning and tumour like processes were identified. A lumbar puncture was never performed on any of these patients. They were transferred to neurosurgery departments for brain biopsy. Diagnosis was achieved based on histology and culture results.

Symptom duration before admission ranged greatly with a median of 14 days in the Danish patients and 20 days in the immigrant group.

The tuberculin skin test was not performed routinely in all patients; only 20 results were available for this study; 15 (75%) were positive. BCG scar status had not been noted in any of the reviewed patient files.

### Cerebrospinal fluid characteristics

A total of 42 patients had a lumbar puncture performed at the time of admission. The remaining eight patients all had tuberculomas and did not have lumbar puncture performed due to symptoms of increased intra-cranial pressure. The white cell count (WCC) was elevated in 38 patients (90%) with lymphocyte predominance in 66%. Protein levels were elevated in 36 patients (86%). Eleven patients had a protein level above 3 g/L. The glucose content was below the minimum reference value in 20 patients (48%). CSF:blood glucose ratios could be calculated in a total of 26 patients; this ranged from 0,09 - 0,71. The ratio was below 0,3 in 50% of these patients (Table 2). Eight out of the nine fatal cases had pleocytosis and elevated protein levels. Only one of the fatal cases had a normal WCC but the CSF had elevated protein content; diagnosis was in this case established by a positive CSF culture.

**Table 2:** Cerebrospinal fluid results in 42 patients

Variable	Normal range	Danes (n = 10)	Immigrants (n = 32)
WCC × 10^6^/L, median (range)	0-4	217 (48-590)	104 (1-2970)
CSF lymphocytes, median (range)		70 (3-98)	76 (4-99)
CSF protein g/L, median (range)	0.2-0.5	0.91 (0.15-3.38)	1.65 (0.1-11.7)
CSF glucose mmol/L, median (range)	2.2-3.9	2.1 (0.7-3.9)	2.2 (0.7-8.7)
CSF:blood glucose ratio, median (range)*	0.33-0.66	0.30 (0.10-0.49)	0.31 (0.09-0.71)
			
Positive smear (%)		0	4 (13)
Positive NAA (%)**		2 (33)	13 (43)
Negative NAA (%)**		4 (67)	17 (57)
Positive mycobacterial culture (%)		7 (70)	24 (75)

WCC = white cell count; CSF = cerebrospinal fluid; NAA = nucleic acid amplification

*Data from 7 Danes and 19 immigrants

** Data from 6 Danes and 30 immigrants

### Radiology

All patients were investigated with either cranial CT or MR scans at time of admission; 22 patients had both scans performed (Table 3). Cranial MR scans showed basal meningeal enhancement in six Danish patients and in 10 immigrants. Tuberculomas were more frequently found in the immigrant patient group. Eight patients with normal CT scans went on to have a cranial MR scan performed and this contributed diagnostically in showing basal meningeal enhancement in all. Eight patients had the combined finding of hydrocephalus and fresh infarct on both CT and MR scans. Only one patient presented both a normal CT and MR scan.

**Table 3:** Neuroradiological findings of 22 patients where both MR & CT was performed

	Danes (n = 7)		Immigrants (n = 15)	
Finding	CT (%)	MR (%)	CT (%)	MR (%)
Basal enhancement	0	6 (86)	2 (13)	10 (67)
Tuberculoma	1 (14)	1 (14)	4 (27)	2 (13)
Hydrocephalus	2 (29)	1 (14)	3 (20)	2 (13)
Fresh cerebral infarct	2 (29)	3 (43)	3 (20)	2 (13)
No pathology	3 (43)	0	6 (40)	2 (13)

Chest X-rays were performed in 48 patients and was found abnormal in 26 cases (54%). Concomitant pulmonary TB was microbiologically verified in 23 cases.

### Microbiology

Results from microbiological analyses performed on CSF are shown in Table 2. In 41 patients (82%) the diagnosis was verified by culture. The number of days before 31 CSF cultures became positive ranged between 7 to 46 days (median 20 days). Nucleic acid amplification (NAA) test was positive for *M. tuberculosis *complex in one sample, but the culture remained negative. In five cases, microbiological diagnosis was obtained through cultures of biopsies from tuberculomas. Furthermore, five patients had positive cultures of non-cerebral specimens (four respiratory and one tissue biopsy from lumbar vertebrae). In eight patients (16%) the diagnosis could not be verified by culture.

Drug susceptibility testing for first line drugs was performed for all 41 culture positive cases. Two isolates were resistant to isoniazid (4%) and one isolate (2%) had resistance to both rifampicin and isoniazid, thus multi-drug resistance. No other polyresistance or extensively drug resistant isolates was identified.

### Treatment and outcome

All patients with fully susceptible isolates were treated with rifampicin (10 mg/kg, max 600 mg), isoniazid (5 mg/kg, max 300 mg), ethambutol (20 mg/kg, max 1200 mg), and pyrazinamide (30 mg/kg, max 2000 mg) in the first 2 months of the intensive phase of treatment followed by rifampicin and isoniazid in the continuation phase of 7- 10 months, except one department where all patients (n = 25) in this latter phase were treated for a minimum of 4 months.

Rifabutin was replaced with rifampicin in one HIV positive patient because a protease inhibitor was part of the anti-retroviral treatment. Rifampicin was discontinued in one patient due to persistent thrombocytopenia; isoniazid was discontinued in one patient due to hepatotoxicity. Two patients did not receive ethambutol as they had received a kidney transplant. In these cases either ofloxacin or moxifloxacin were included in the treatment. Fluoroquinolones and amikacin were included in the treatment of the patient with multi-drug resistant TB. Treatment was then given for 24 months.

Corticosteroids were used in all but six patients, of whom three died before treatment was initiated. Prednisolone was generally used at an initial dose of 1 mg/kg with a gradual reduction over 4-6 weeks. There were 12 paediatric patients and all but two received prednisolone; one died before TBM was diagnosed.

Neurosurgical intervention in terms of shunting was performed in four patients with altered cerebral condition and severe hydrocephalus identified on MR or CT scans.

The delay in initiation of treatment post-admittance ranged from 0 to 52 days with the highest median of 7 days found in the Danish patient group.

All patients, except two, who were transferred to their home country, completed the treatment. 16 patients (33%) had full recovery and 23 (48%) patients had sequelae. A total of nine patients (19%) died; the age range was 4-71 years (median 45 years).

Three patients died after several months' treatment due to severe neurological sequelae. The remaining six patients died within one month of admittance; three of these patients never received any treatment. The cause of death in the six patients was severe hydrocephalus and infarcts.

## Discussion

Extrapulmonary TB accounts for 1/3 of all TB notifications in Denmark. In 2008, 60% of notified cases were of other ethnic background than Danish [2]. TBM remains a rare manifestation of extrapulmonary TB in Denmark with a consistent incidence of five to six new cases per year during the past ten years. Almost 80% of our study population consists of immigrants from countries of high tuberculosis endemicity. This resembles a significant increase from the figure of 26% reported in a Danish study from the 1970s-1980s [6] and also marks a slight increase from the most recent Danish study where this figure was 65% [7]. The predominance of immigrants cannot be linked to health disparity, since the Danish health system has free and equal access for all citizens. Another important characteristic of our patient population is the high prevalence of underlying immunosuppressive disease; a total of 64% are found in this study, compared to recent figures of 25% - 35% [6,7]. 10% of the patients were HIV positive, but it is worrying that HIV serostatus was not available in 42%. Since active TB is more common in people infected with HIV [8], it must be stressed that HIV testing should always be performed in conjunction with diagnosing TBM.

The clinical presentation of TBM is vague with non-specific symptoms that are hard to distinguish from other types of bacterial meningitis. It is noted that less than half our patients had the typical sign of meningeal stiffness. A long history of illness (over 5-6 days) has previously been shown to be a clinical variable highly predictive of TBM [9,10]; we have similar findings with a median of 10 and 14 days respectively in our two patient populations.

Biochemical CSF analysis revealed that the majority of patients (66%) had the characteristic findings of pleocytosis with lymphocyte predominance. Also, protein levels were, as would be expected, elevated in the majority of patients as well. Previous studies from Iran, India and Vietnam have found the percentage of CSF lymphocytes to be one of the strongest diagnostic variables predictive of TBM, especially when the total WCC is less than 1000 × 10^3^/ml [11,12]. Only one of our patients had a WCC above 1000 × 10^3^/ml.

Direct microscopy of CSF for acid-fast bacilli was positive in only 9.5% of the specimens. This low sensitivity is unfortunate, as this is the simplest diagnostic tool available and can also be used easily in a low-resource setting. Other series have found varying sensitivities of a direct smear and figures as high as 58% have previously been reported [13]. NAA tests have been shown to be more sensitive and specific than microscopy in diagnosing pulmonary TB [14], but their performance in extrapulmonary specimens, such as CSF, have been disappointing [15]. In this study the reported 42% sensitivity of NAA is considerably low, but still better than the sensitivity of smears. This shows that diagnosing TBM in a setting of high resourcefulness remains challenging.

The culture of CSF samples was positive for *M. tuberculosis *in 74%; this figure is higher than the 55% reported in two previous Danish studies [6,7]. Culture remains an essential, but time consuming, tool in diagnosing TBM; we report a median of 20 days for bacterial growth to be confirmed. Culture can verify the TBM diagnosis but cannot be relied on in the initial, critical diagnostic phase. Culture is however still of importance, especially for identifying resistant isolates.

The most common findings on neuroradiological images were basal meningeal enhancement and tuberculomas. As seen clearly in especially the Danish group of patients, MR scans proved more sensitive for identifying meningeal enhancement than CT scans (86% vs. 0%). Tuberculomas were more commonly found in the immigrant group. Cranial CT scans seem to be just as sensitive as MR scans in identifying hydrocephalus, infarcts and tuberculomas. A recent study found that MR is superior to CT in identifying basal meningeal enhancement as well as infarcts; hydrocephalus was in the same study detected equally by MR and CT scans [16]. In conclusion, MR scans should be considered as the primary choice for neuroradiological imaging in the initial diagnostic phase in a high-resource setting. A total of 46% of our patients also had pulmonary TB and this emphasises the importance of chest X-rays and microbiological analyses on respiratory specimens in the diagnostic process.

The treatment regimes described in this study are not homogenous. Standard anti-tuberculous drugs were used at all centres, but the total length of treatment varied from 6-12 months. National guidelines suggest treatment duration of 6 months, except in severe cases, where 9-12 months treatment regimens are applied, as also recommended by WHO [17]. This is in contrast to the recommendations applied in the United Kingdom and the United States, where all TBM patients are treated for 9-12 months [18,19]. A high proportion of the patients were treated with adjuvant prednisolone treatment; this is a well-established component of TBM treatment and has been shown to significantly reduce mortality rates [20].

The mortality rate was 19%. The cause of death was hydrocephalus and/or brain damage in all patients. Similar studies from other developed nations such as the United States and Australia have reported mortality rates of 41% [21] and 7% [22], respectively. From countries of endemic TB as well as high HIV prevalence, mortality rates have been significantly higher: from South Africa 69% [23] and from Vietnam 67% [24]. The proportion of patients with various neurological sequelae in our study is high at 50%, consistent with previous reports [7].

## Conclusions

TBM remains a serious disease even in a setting of low TB incidence such as Denmark. The disease primarily affects immigrants from regions of high TB endemicity and has a high mortality rate and a high rate of sequelae among survivors. The diagnosis of TBM remains difficult, even in a high-resource setting, as the currently available diagnostic tools lack sensitivity. Although TBM only affects a handful of people each year in Denmark, the clinician must be prepared to treat empirically if the suspicion of TBM has arisen.

## Competing interests

The authors declare that they have no competing interests.

## Authors' contributions

AC and IJ collected and analysed patient data and drafted the article. PA provided the notification data and VT provided microbiological data. IJ and ÅA supervised the study. All authors read and approved the manuscript.

## Pre-publication history

The pre-publication history for this paper can be accessed here:

http://www.biomedcentral.com/1471-2334/11/47/prepub

## References

[B1] Phypers M Harris T Power C CNS tuberculosis: a longitudinal analysis of epidemiological and clinical features Int J Tuberc Lung Dis 2006 10 1 99 103 16466045

[B2] Andersen PH Tuberkulose 2008 del 1 Epi News 2009 No 50, Statens Serum Institut, Denmark

[B3] Poulsen S Roenne T Kok-Jensen A Bauer J Miorner H Epidemiology of tuberculosis in Denmark 1972-1996 Ugeskr Laeger 1999 161 3452 7 10388353

[B4] Samuelsson S Tuberkulose 2000 del 1 Epi News 2001 No 43, Statens Serum Institut, Denmark

[B5] Lillebaek T Andersen AB Bauer J Dirksen A Glismann S de Haas P Risk of Mycobacterium tuberculosis transmission in a low-incidence country due to immigration from high-incidence areas J Clin Microbiol 2001 39 3 855 61 10.1128/JCM.39.3.855-861.2001 11230395 PMC87841

[B6] Jensen TH Magnussen P Eriksen NHR Skinhøj P Tuberculous meningitis: 23 Cases from a 12-year period (1976-1987) Dan Med Bull 1990 37 459 62 2272213

[B7] Bidstrup C Andersen PH Skinhøj P Andersen AB Tuberculous meningitis in a Country with a Low Incidence of Tuberculosis: Still a Serious Disease and a Diagnostic Challenge Scand J Infect Dis 2002 34 811 14 10.1080/0036554021000026938 12578148

[B8] Getahun H Gunneberg C Granich R Nunn P HIV infection-associated tuberculosis: the epidemiology and the response Clin Infect Dis 2010 50 201 7 10.1086/651492 20397949

[B9] Thwaites GE Chau TT Stepniewska K Phu NH Chuong LV Sinh DX Diagnosis of adult tuberculous meningitis by use of clinical and laboratory features Lancet 2002 360 9342 1287 92 10.1016/S0140-6736(02)11318-3 12414204

[B10] Kumar R Singh SN Kohli N A diagnostic rule for tuberculous meningitis Arch Dis Child 1999 81 3 221 4 10.1136/adc.81.3.221 10451394 PMC1718078

[B11] Moghtaderi A Alavi-Naini R Izadi S Cuevas LE Diagnostic risk factors to differentiate tuberculous and acute bacterial meningitis Scand J Infect Dis 2009 41 3 188 94 10.1080/00365540902721384 19235095

[B12] Youssef FG Afifi SA Azab AM Wasfy MM Abdel-Aziz KM Parker TM Differentiation of tuberculous meningitis from acute bacterial meningitis using simple clinical and laboratory parameters Diagn Microbiol Infect Dis 2006 55 4 275 8 10.1016/j.diagmicrobio.2006.01.027 16626906

[B13] Thwaties GE Chau TT Farrar JJ Improving the bacteriological diagnosis of tuberculous meningitis J Clin Microbiol 2004 42 378 79 10.1128/JCM.42.1.378-379.2004 14715783 PMC321694

[B14] Johansen IS Thomsen VØ Johansen A Andersen P Lundgren B Evaluation of a new commercial assay for diagnosis of pulmonary and nonpulmonary tuberculosis Eur J Clin Microbiol Infect Dis 2002 21 6 455 60 10.1007/s10096-002-0737-x 12111602

[B15] Pai M Flores LL Pai N Hubbard A Riley LW Colford JM Jr Diagnostic accuracy of nucleic acid amplification tests for tuberculous meningitis: a systematic review and meta-analysis Lancet Infect Dis 2003 3 10 633 43 10.1016/S1473-3099(03)00772-2 14522262

[B16] Pienaar M Andronikou S van Toorn R MRI to demonstrate diagnostic features and complications of TBM not seen on CT Childs Nerv Syst 2009 25 941 7 10.1007/s00381-008-0785-3 19107489

[B17] World Health Organization Treatment of Tuberculosis Guidelines http://whqlibdoc.who.int/publications/2010/9789241547833_eng.pdf

[B18] American Thoracic Society Treatment of tuberculosis Am J Respir Crit Care Med 2003 167 603 662 10.1164/rccm.167.4.603 12588714

[B19] British Thoracic Society Chemotherapy and management of tuberculosis in the United Kingdom: recommendations 1998 Thorax 1998 53 536 48 10.1136/thx.53.7.536 9797751 PMC1745276

[B20] Prasad K Singh MB Corticosteroids for managing tuberculous meningitis Cochrane Database Syst Rev 2008 1 CD002244 18254003 10.1002/14651858.CD002244.pub3

[B21] Porkert MT Sotir M Parrott-Moore P Blumberg HM Tuberculous meningitis at a large inner-city medical center Am J Med Sci 1997 313 6 325 31 10.1097/00000441-199706000-00002 9186145

[B22] Kent SJ Crowe SM Yung A Lucas CR Mijch AM Tuberculous Meningitis: A 30-Year Review Clin Infect Dis 1993 17 987 94 8110957 10.1093/clinids/17.6.987

[B23] Karstaedt AS Valtchanova S Barriere R Crewe-Brown HH Tuberculous meningitis in South African urban adults Q J Med 1998 91 743 747 10.1093/qjmed/91.11.743 10024937

[B24] Torok ME Chau TTH Mai PP Phong ND Dung NT Chuong LV Clinical and Microbiological Features of HIV-Associated Tuberculous Meningitis in Vietnamese Adults PLoS ONE 2008 3 3 e1772 10.1371/journal.pone.0001772 18350135 PMC2262136

